# Some Remarks on Bacteriology and Its Relation to Scientific Medicine*Read before the S. C. Medical Association.

**Published:** 1891-04

**Authors:** A. Bethune Patterson

**Affiliations:** Late Assistant to the Royal London Ophthalmic Hospital and Golden Square Throat Hospital, London, England; Atlanta, 27 Old Capitol


### SOME REMARKS ON BACTERIOLOGY AND ITS RELATION TO SCIENTIFIC MEDICINE.*

BY A. BETHUNE PATTERSON, M. D., LATE ASSISTANT TO THE ROYAL LONDON OPHTHALMIC HOSPITAL AND GOLDEN SQUARE THROAT HOSPITAL, LONDON, ENGLAND.

*Read before the S. C. Medical Association.

That bacteria is the immediate cause of many diseases, is not only generally accepted, but a fact so thorougly established, that a knowledge of the science and art becomes indispensable to a thorough understanding of modern pathology. The startling success as met with in the surgical wards to-day—are due to the germ theory of disease—many of the failures met with are due to a lack of training in the technic of antiseptic surgery, and a deficiency in a knowledge of the principles which underlie this rapidly growing science; a science which has so engrafted itself into State medicine, and on pathology, as to have forced upon the medical teachers of our country the necessity of practical instruction in bacteriology.

I have read somewhere, that our statistics show that during the last decade, the death rate has diminished one-half. This great saving of life has not been so much due to a more thorough knowledge of therapeutic agencies, as to preventive measures—measures that were suggested after an investigation into the life history of bacteria.

Time will not admit of a thorough or extensive discussion of the subject. I will be content to briefly give the result of my investigation into the species of bacteria, their origin, structure, development, vegetating condition, their cultivation and the influence they exert on the tissue.

The word bacteria means a rod, the term is now applied to some of the minute forms of animal and vegetable life. These living organisms called schigomycitus are popularly called bacteria and micrococci. There is a relationship that exists between these bacteria, similar to that which exists between the various species of animal and vegetable life, of larger development, that do not require a magnifying glass in studying their form and habits.

Those bacteria which require similar nutrition and elementary alkaloids, resemble each other and produce symptoms in the economy, so analogous as to be easily classified. Owing to their small size, it has been impossible to investigate their structure, but they certainly pursue the same mechanical course that cells do in multiplying—supposing the structure of some of them to be the same as in all cell bodies, from the effect that certain dyes have upon them.

Species are frequently determined by their color, thus some are brown, others violet, green, blue, yellow and red. From what part of the bacteria this color is reflected, has not been determined. Some of the larger ones, when properly acted upon by a staining preparation, show a membrane and protoplasm.

Motor appendages have been sought for, and it is supposed by some, that their motion is due to cilia, which appear to be organs of motion in certain cells.

The Oscellatoriæ are large forms, found among the lower organisms, possessing motion, though no organs of motion have been found in them. The free motion of bacteria is beautiully shown in a drop of urine, that has been standing for some time exposed to the air.

These microscopical beings assume a variety of forms; those seen in relapsing fever present a cork-screw appearance, and are termed spirilla. Erysipelas, pneumonia and gonorrhœa furnish typical forms of cocci; tuberculosis, diphtheria and syphilis, the rod-forms; under certain circumstances, deviations in form do occur. It has never yet been shown that a bacterium appears at one time as a genuine micrococcus, at another as a bacillus, and then again as a spirilla. The changes that have been observed to have taken place in the organism of Finkler, have also been observed in the cholera germ.

It must be admitted that in the cultivation, the typical forms invariably appeared, and the changes are only variations upon that typical form. DeBarry divided bacteria into two classes —endsporous and anthrosporous. In tracing the development of these vegetating bacteria, we find in the former the straight rod-forms, called baccillus, and some of the spirilla. These cells are motionless, and develop rapidly, directly this process is discontinued, and the spore begins to form; first a minute granule begins to appear in the protoplasm, and soon assumes proportions of round or elongated bodies, reaching its full growth in a short time. As the spore develops, the contents of the cell-protoplasm etc., disappear. DeBarry thinks the protoplasm is consumed by the spore as nutrition. The spore has its own membrane, and is surrounded by the membrane of the mother cell, which soon disappears. When a full grown spore is placed in an atmosphere possessing necessary elements of nutrition, moisture, and sufficient degree of heat, germination begins. It then soon loses its characteristic appearance, and reaches the shape and dimensions of mother cell.

Athrospore is a transformation of an entire part or cell of a bacterium—the membrane of the bacterium forming the membrane of the spore. DeBarry says: "We cannot say that facts will not come to light in course of time, which will do away with any sharp separation between the two divisions; these forms are similar in every respect, except as to their spore formation.

Cultivation and isolation of bacteria require some experience and delicacy of manipulation. This becomes essential to the pathologist who wishes to know something of the inner life of these interesting beings. They display some degree of fastidiousness as to the character of their food and abiding place. Unless these are in every way congenial, they remain inactive, or else perish. We are not surprised at this, when we reflect upon the predilections of higher forms of vegetation. It has been observed that germs that thrive and grow rapidly upon one form of potato are wholly inactive upon others. Fluids and solid substances are used as soils in which to grow germs, of the former, beef tea, chicken broth, infusion of hog blood, serum, are the usual selections. But these present serious disadvantages in getting pure cultures. Solids are more popular, and attended with less difficulty in isolating bacteria.

Koch first used the common potato. In making microscopical observations of cultures, fluids are generally employed, and in making the selection special attention must be given to its transparency. Gelatinous substances, such as are transparent, furnish a medium that is very desirable, and in making observations upon cultures in these, the chances of mixing are much diminished. Agar-agar is much used by Koch; a small quantity of this vegetable jelly is added to peptonized meat juice, and heated until it liquifies, this is poured into glass tubes about one-third full, and then stopped up with cotton, which acts as a barrier to the entrance of germs from the atmosphere. Tubes are sterilized by boiling in a water bath for several minutes daily for several days.

Knives, needles, and other instruments must be carefully sterilized before commencing an experiment. A wire or needle is used for introducing the micro-organisms into or on the surface of the culture medium, and every care must be used to prevent the introduction of germs from without. The tube is now placed in an incubator, where a given temperature is maintained After a while there will be objective signs of growth, viz., a turbidity along the track of the needle—species may be recognized by the color they reflect. The degree of temperature and the length of time taken in developing are important and significant points to note.

To isolate bacteria, the plate method, as used by Koch, is very ingenious. The needle is introduced into the strata, the color of which indicates the species you desire to cultivate, and a sterilized medium is again inoculated, placed in an incubator, and these manipulations are gone through with as many times as is necessary to separate the bacteria, in other words, to get a pure culture. These micro-organisms are in a position to be observed under the microscope. Glass slides, with cells ground in them, are very convenient for cultivating, and observing, from day to day, the growth of bacteria. It is not intended to go into a thorough description of the technic of bacteria culture.

I shall now consider briefly the connection of bacteria with pathological conditions—their introduction into the tissue, and their behavior there. Their effects upon the constitution are both local and general. Micro-organisms of a specific character probably find their way into the system through the air-passages, abrasions of the skin and mucous surfaces, and by way of the digestive elements.

Bacteria seem to have a predilection for tissue and blood-cells. There is an antagonism existing between micro-organisms and these little bodies. The hypertrophied cells observed by Paine are possibly due to nutrition furnished by vanquished micro-organisms, or to hyperaemia, which he claims always results; he says there is "a marked increase in the size of elements characteristic of chronic bacterial inflammation."

Bacteria, when in a medium suitable to their germination, propagate themselves very rapidly. The disturbance set up in the constitution by this process is due not only to the organisms themselves, but also to the poisons they generate, called ptomaines. Vaughn's experiments show that only a few of these animal alkaloids are poisonous; a great many are non-poisonous or inert, as their progenitors are powerless to bring about any pathological condition.

Micro-organisms, to produce ptomaines, must be placed in a suitable medium, surrounded by all the conditions necessary for their special action. Briegeo's experiments prove the above fact; thus the typhoid bacillus, when placed in beef-tea, grows and develops ptomaines; the same vegetating process went on in a solution of peptone—yet there were no ptomaines found.

In conclusion it will not be amiss to allude briefly to some of the methods employed in recognizing bacteria. Freedlander says: "Just as there is no one general method of histological examinations, so we cannot expect to discover any single method of demonstrating micro-organisms." There are a number of new methods of staining bacteria. I shall allude to a few that I have had some experience with. For instance, we desire to exhibit the tubercle bacilli, the first step would be to place a drop of the suspected matter between two covered glasses, and press them together so as to make the film as thin as possible. After drying by passing a few times through the flame of a spirit lamp, they are ready for the staining solution.

Bacteria have a remarkable affinity for the analine dyes ; gentian violet, fuchsine, are ordinarily used. Lay the cover glass in the dye, (say gentian violet) sputum side down and let them float, it will take about one-half to one hour to stain them, under an elevated temperature of 110 deg. or 120 deg., after which they are washed off with alcohol to rid them of surplus staining. Then place them in a solution of alcohol and nitric acid, observe the effect, should not be allowed to remain over eight minutes, removing them as soon as the color is gone out of the mass of sputa. The tubercle bacilli retains the color, while everything else is discolored. They are ready to be mounted after drying them with filter paper. If contrast color or double staining is desired, place the specimens treated as above in a saturated aqueous solution of Bismork Brown. Carbolized fuchsine stains the bacteria red, and as a contrast color, nuthlyne blue may be used.

I do not believe physicians generally appreciate the importance of acquiring a knowledg of bacteriology. as it relates to diagnosis, prognosis and treatment. The Northern and Western colleges are establishing laboratories, offering every facility to physicians and students of acquiring a knowledge of this science and art. The time is not far distant when our Southern schools will follow their example.

*Atlanta, 27 Old Capitol.*

